# SeriesSleepNet: an EEG time series model with partial data augmentation for automatic sleep stage scoring

**DOI:** 10.3389/fphys.2023.1188678

**Published:** 2023-08-28

**Authors:** Minji Lee, Heon-Gyu Kwak, Hyeong-Jin Kim, Dong-Ok Won, Seong-Whan Lee

**Affiliations:** ^1^ Department of Biomedical Software Engineering, The Catholic University of Korea, Bucheon, Republic of Korea; ^2^ Department of Artificial Intelligence, Korea University, Seoul, Republic of Korea; ^3^ Department of Brain and Cognitive Engineering, Korea University, Seoul, Republic of Korea; ^4^ Department of Artificial Intelligence Convergence, Hallym University, Chuncheon, Republic of Korea

**Keywords:** automatic sleep stage scoring, convolutional neural network, bi-directional long-short term memory, single-channel EEG, deep learning

## Abstract

**Introduction:** We propose an automatic sleep stage scoring model, referred to as SeriesSleepNet, based on convolutional neural network (CNN) and bidirectional long short-term memory (bi-LSTM) with partial data augmentation. We used single-channel raw electroencephalography signals for automatic sleep stage scoring.

**Methods:** Our framework was focused on time series information, so we applied partial data augmentation to learn the connected time information in small series. In specific, the CNN module learns the time information of one epoch (intra-epoch) whereas the bi-LSTM trains the sequential information between the adjacent epochs (inter-epoch). Note that the input of the bi-LSTM is the augmented CNN output. Moreover, the proposed loss function was used to fine-tune the model by providing additional weights. To validate the proposed framework, we conducted two experiments using the Sleep-EDF and SHHS datasets.

**Results and Discussion:** The results achieved an overall accuracy of 0.87 and 0.84 and overall F1-score of 0.80 and 0.78 and kappa value of 0.81 and 0.78 for five-class classification, respectively. We showed that the SeriesSleepNet was superior to the baselines based on each component in the proposed framework. Our architecture also outperformed the state-of-the-art methods with overall F1-score, accuracy, and kappa value. Our framework could provide information on sleep disorders or quality of sleep to automatically classify sleep stages with high performance.

## 1 Introduction

Sleep is associated with human health and quality of life (Siclari and Tononi, 2017). However, millions worldwide suffer from health problems due to sleep disorders (Lee et al., 2019b). Sleep stage classification is an essential step in diagnosing or treating sleep disorders (Phan et al., 2019). Polysomnography (PSG) signals are widely used in sleep measurement examinations. These signals include biosignals from electroencephalography (EEG), electrooculography (EOG), electromyography (EMG), and electrocardiography (ECG) (Fan et al., 2021). Specifically, EEG signals are extensively used as unimodal biosignals to analyze sleep quality and classify sleep stages.

Each sleep stage has a distinct characteristic (Fu et al., 2021). These sleep stages are divided into either the Rechtschaffen and Kales (R&K) standard (Wolpert, 1969) or the American Academy of Sleep Medicine (AASM) standard (Grigg-Damberger, 2009). The R&K standard separates sleep into six stages: wakefulness (Wake), rapid eye movement (REM), and four sleep stages (S1–S4) as non-rapid eye movement (NREM). The AASM divides sleep stages into five stages. The Wake and REM stages remained the same as in R&K; however, the NREM stage differed. During the S3 stage, delta activity appears, and in S4, it is present for more than 50% of the time (Sinton and McCarley, 2004). In the AASM, S3 and S4 stages are represented as a single sleep stage, the N3 stage. In the N3 stage, which is called slow-wave sleep, delta activity appears dominantly (Fu et al., 2021). During the N1 stage, alpha activity decreases and theta activity tends to increase. The N2 stage is the beginning of full-fledged sleep, and unique oscillations called K-complexes and spindles appear in the central region (Choi et al., 2019). In the REM stage at the end of the sleep cycle, theta activity is remarkably similar to the that in N1 stage (Lee et al., 2019a). However, eye movement occurs during the REM stage (Siclari and Tononi, 2017). In the Wake stage, the alpha activity becomes prominent (Grigg-Damberger, 2009).

Sleep experts manually distinguish sleep stages based on the characteristics of each sleep stage. However, this can be tedious and time-consuming (Phan et al., 2018; Ravan and Begnaud, 2019). Hence, automatic sleep stage classification methods have been widely developed. Initially, handcrafted features extracted from biosignals, such as EEG or EOG signals, were used to classify sleep stages (Redmond and Heneghan, 2006). These features are primarily related to the spectral or temporal characteristics of the sleep stages. Nevertheless, these handcrafted features may not be generalized to a larger population owing to the heterogeneity associated with the individuals (Supratak et al., 2017). Deep learning has recently emerged as an alternative method to solve these problems. Its strength is that the model learns the optimal features to be extracted from the input data without humans having to put in the effort of directly designating the features (Kim et al., 2019; Duan et al., 2021). Many studies have begun to use deep learning for sleep stage classification.

The class imbalance problem directly affects the classification performance (Krawczyk, 2016; Thung et al., 2018). In this respect, sleep stage scoring performance is limited owing to the different proportions of sleep stages (Supratak et al., 2017). Two approaches have been used to solve this problem. First, there is a data-level approach that oversamples the data with a small number of classes. This is simple and intuitive to balance the number of classes but has the disadvantage of increasing the likelihood of overfitting and increasing the computational time. It is also sensitive to noise or outliers (Kumar et al., 2021). Another approach is to design a cost function at the algorithm level. The classification of rare classes is penalized more than the incorrect classification of abundant classes. By designing a cost function, it naturally generalizes in favor of a rare class (Kim et al., 2022). Therefore, we approach the algorithm level to solve the class imbalance problem in sleep stage scoring.

In this paper, we propose an automatic sleep scoring model, *SeriesSleepNet*, based on convolutional neural networks (CNN) and bidirectional long short-term memory (bi-LSTM) with partial data augmentation using single-channel EEG signals. In this study, data augmentation was applied using the sliding window method to learn the connected time information in a small series and, not to solve the class balance problem. In addition, it was used in bi-LSTM after CNN, not at the beginning of the framework. Therefore, we used the term “partial data augmentation.” The loss function was modified by adaptively providing additional weights using the training performance to solve the data balance problem. Specifically, we used the F1-score, harmonic mean of precision and recall, for the weighting of the loss function. The proposed framework can provide sleep information for individuals with sleep stage classification for healthcare applications.

The novelty of our paper is as follows:

• We proposed a deep learning model that can learn both inter-epoch and intra-epoch in temporal information suitable for sleep stage classification.
• We proposed a partial data segmentation using the sliding window method to learn inter-epoch temporal information.
• We designed an adaptive cross-entropy loss function that adaptively changes class-wise loss weights based on previous training results to address the class imbalance in the sleep stage.

## 2 Related works

Many studies have been actively conducted on automatic sleep stage scoring, and they either utilize only a single channel for practical purposes or multiple channels for performance. Table 1 shows related studies for each biosignal using deep learning.

**TABLE 1:** Related works using deep learning for automatic sleep stage scoring.

Type	Model	Year	Architecture	Dataset	Input signal	Evaluation method	Performance
Single channel	DeepSleepNet Supratak et al. (2017)	2017	CNN + RNN	Sleep-EDF	1 EEG	20 folds LOSO CV	Acc. 82.0%, F1 76.9%
				MASS-SS3		31 folds CV	Acc. 86.2%, F1 81.7%
	ResnetLSTM Sun et al. (2018)	2018	CNN + RNN	Sleep-EDF	1 EEG	20 folds LOSO CV	Acc. 81.0%, F1 73.6%
	Sors et al. (2018)	2018	CNN	SHHS-1	1 EEG	50(train)/20(valid)/30(test)	Acc. 86.8%, F1 78.0%
	Yildirim et al. (2019)	2019	CNN	Sleep-EDF	1 EEG	70(train)/15(valid)/15(test)	Acc. 90.8%, F1 76.0%
	U-time Perslev et al. (2019)	2019	CNN	Sleep-EDF	1 EEG	20 folds LOSO CV	F1 79.0%
	SleepEEGNet Mousavi et al. (2019)	2019	CNN + RNN	Sleep-EDF	1 EEG	20 folds CV	Acc. 84.3%, F1 79.7%
	IITNet Seo et al. (2020)	2020	CNN + RNN	Sleep-EDF	1 EEG	20 folds LOSO CV	Acc. 83.6%, F1 76.5%
				MASS-SS3		31 folds CV	Acc. 86.2%, F1 80.0%
				SHHS-1		50(train)/20(valid)/30(test)	Acc. 86.3%, F1 78.8%
	AttnSleep Eldele et al. (2021)	2021	CNN	Sleep-EDF	1 EEG	20 folds LOSO CV	Acc. 84.4%, F1 78.1%
				SHHS-1		20 folds CV	Acc. 84.2%, F1 75.3%
	DeepSleepNet-Lite Fiorillo et al. (2021)	2021	CNN	Sleep-EDF	1 EEG	20 folds LOSO CV	Acc. 86.1%, F1 79.6%
	SleepFCN Goshtasbi et al. (2022)	2022	CNN	Sleep-EDF	1 EEG	20 folds LOSO CV	Acc. 84.8%, F1 78.8%
				SHHS-1		20 folds CV	Acc. 81.3%, F1 72.0%
Multi channel	Chambon et al. (2018)	2018	CNN	MASS-SS3	6 EEG + 2 EOG + 3 EMG	5 folds CV	Acc. 77.6, F1 72.2%
	MultitaskSleepNet Phan et al. (2018)	2018	CNN	Sleep-EDF	1 EEG + 1 EOG + 1 EMG	20 folds LOSO CV	Acc. 82.3%, F1 74.7%
				MASS (SS1 ∼SS5)		20 folds CV	Acc. 83.6%, F1 77.9%
	SeqSleepNet Phan et al. (2019)	2019	CNN + RNN	MASS (SS1 ∼SS5)	1 EEG + 1 EOG + 1 EMG	20 folds CV	Acc. 87.1%, F1 83.3%
	SleepPrintNet Jia et al. (2020)	2020	CNN	MASS-SS3	6 EEG + 2 EOG + 3 EMG	31 folds CV	Acc. 88.8%, F1 84.3%
	Olesen et al. (2021)	2021	CNN + RNN	SHHS (1 and 2)	1 EEG + 2 EOG + 1 EMG	87.5(train)/2.5(valid)/10(test)	Acc. 87.2%
	RobustSleepNet Guillot and Thorey (2021)	2021	RNN	Sleep-EDF	2 EEG + 1 EOG	5 folds CV	F1 78.8%
				MASS-SS3	3 EEG + 2 EOG+1 EMG	5 folds CV	F1 82.2%
				SHHS-2	2 EEG + 1 EOG+1 EMG	3 folds CV	F1 79.2%
	U-sleep Perslev et al. (2021)	2021	CNN	Sleep-EDF	1 EEG + 1 EOG	75(train)/10(valid)/15(test)	F1 79.0%
				SHHS-1			F1 80.0%
				MASS-SS3			F1 80.0%
	CoSleepNet Efe and Ozsen (2023)	2023	CNN + LSTM	Sleep-EDF	1 EEG + 1 EOG	20 folds LOSO CV	Acc. 81.1%, F1 79.8%

Cross-validation = CV; Leave-one-subject-out = LOSO; Montreal archive of sleep studies = MASS; The sleep heart health study = SHHS.

### 2.1 Using single-channel EEG signals

DeepSleepNet (Supratak et al., 2017) was proposed as an end-to-end deep learning model and comprised two CNNs for feature extraction and bi-LSTM as a classifier. The kernel size of each CNN was set differently to extract both time series and frequency-domain information. The reported overall accuracy was 86.2% for the F4-EOG channel of the MASS dataset and 82.0% for the Fpz-Cz EEG channel of the Sleep-EDF dataset.

Another model (Sors et al., 2018) using CNN was designed. This model was composed of 12 convolutional layers, followed by one or two fully connected layers. A softmax regression layer was then used to predict the class probabilities. Four epochs were concatenated into one sample to consider the surrounding epochs. The reported overall accuracy was 87.0% for the C4-A1 EEG channel in the SHHS dataset.

One model (Yildirim et al., 2019) comprised two CNNs, and one max-pooling layer was repeated five times. The reported overall accuracy was 90.83% for the Fpz-Cz EEG channel of the Sleep-EDF dataset. However, unlike other papers that include 30 m before and after sleep as Wake, the reported classification performance included all waking hours. The number of Wake epochs is overwhelmingly higher than in other sleep stages, which affects high performance, making the results difficult to generalize. It is usually recommended to classify the sleep stages, including the sleeping periods and 30 m before and after sleep (Tsinalis et al., 2016). Therefore, it is challenging to compare the performance of the proposed model to other models.

U-time (Perslev et al., 2019) was developed using a temporal fully convolutional network. This model comprises an encoder block, a decoder block, and a segment classifier. The encoder block repeats a structure with one max-pooling layer following two convolutional layers four times. The decoder block comprises four transposed-convolution blocks, each of which performs nearest-neighbor upsampling of its input followed by convolution and batch normalization. Finally, the sleep stages are predicted from the segment classifier that performs average-pooling and convolutional computation. The reported global F1-score was 79.0% for the Fpz-Cz EEG channel of the Sleep-EDF dataset.

AttnSleep (Eldele et al., 2021) was designed based on a multi-resolution CNN and adaptive feature recalibration. Moreover, this framework adds a temporal context encoder and proposes a class-aware loss function to solve the class imbalance problem. They achieved an overall accuracy of 84.4% for the highest performance using the Sleep-EDF dataset. However, the proposed weighted loss function is based on the distribution of each class and does not include the performance per class in the weight.

Others (Goshtasbi et al., 2022) designed an automatic sleep scoring model with a full CNN named SleepFCN. SleepFCN consists of two main modules: a multi-scale feature extractor and a temporal sequential extractor. Each module is based on multiple convolutional layers. In the multi-scale feature extractor module, the model uses two different sizes of convolutional kernels (large and small) to effectively extract the features from various frequency bands, considering the time-frequency resolution trade-off. In the temporal sequential extractor module, the authors used dilated causal convolutional layers to efficiently capture the sequential features of the sleep transition. They also used the weighted cross-entropy loss function to alleviate the class imbalance problem in sleep stage scoring, based on the ratio between the minor and major classes. Using this approach, the authors achieved a faster and simpler model architecture with high performance compared to recurrent neural networks (RNN)-based models.

Sleep stage scoring frameworks using single-channel signals have become more practical, but classification performance is not as high as that of multi-channel signals. Therefore, an algorithm is required to improve performance using only single-channel EEG signals.

### 2.2 Using multi-channel signals

EEG can also be used to classify multi-channels for sleep stages. One study utilized both the Pz-Oz channel and the FPz-Cz channel of the EEG signal from the PSG signals (Sharma et al., 2021). Discrete wavelet transform was used to decompose a 30-s EEG segment to represent each epoch as a simple subband signal related to a specific frequency, and dispersion entropy was used to quantify signal uncertainty. As a result, in the Sleep-EDF dataset, 86.73% accuracy was obtained in the 5-class sleep stage classification using a random forest classifier. However, most multi-channel studies used EEG signals and other biosignals together.

CNN model (Chambon et al., 2018) proposed a deep learning architecture to perform temporal sleep stage classification using multivariate and multimodal time series. The model is composed of three convolutional layers and two max-pooling layers. The reported overall accuracy was approximately 80.2% for 20 EEG channels and 2 EMG channels in the MASS dataset.

MultitaskSleepNet (Phan et al., 2018) is a joint classification and prediction CNN framework. This comprises two CNNs and two max-pooling layers. Subsequently, the multitask softmax layer classifies and predicts the sleep stages of each epoch. The reported overall classification accuracy was 82.3% for the Fpz-Cz EEG and horizontal EOG channels of the Sleep-EDF dataset and 83.6% for the C4-A1 EEG, average EOG (ROC-LOC), and average EMG (CHIN1-CHIN2) channels of the MASS dataset. They proposed a sequence-to-sequence automatic sleep stage classification model SeqSleepNet (Phan et al., 2019). The second model comprises two bidirectional RNNs. The first bi-RNN extracts features from each epoch, and the second bi-RNN predicts a label by considering sequential information. The authors used gated recurrent unit cells for the bi-RNN owing to their computational efficiency. In addition, the short-time Fourier transform was used to convert the raw input signals into temporal-spectral images. As a result, SeqSleepNet represented the highest performance in the N1 and REM stages compared to previous methods. The reported overall accuracy for the C4-A1 EEG, average EOG (ROC-LOC), and average EMG (CHIN1-CHIN2) channels of the MASS dataset was 87.1%.

Chriskos *et al.* (Chriskos et al., 2019) designed a classification framework based on CNN using cortical connectivity images. Synchronization features derived from cortical interactions are used as the input data. They performed an independent component analysis to remove noise components related to body movements, and interference from other biological signals such as EOG, EMG, and ECG modulation. The extracted functional connectivity metrics are then processed as multidimensional images, that are the input for CNN. The reported overall accuracy was 99.85% for 19 EEG, two EOG, one ECG, and one EMG channels of the experimental data from the German Aerospace Agency.

RobustSleepNet (Guillot and Thorey, 2021) shows robust performance across various unseen sleep datasets. To ensure the robustness of the model, the authors mainly focused on handling the incompatibility of the input data shape and extracting input invariant features. RobustSleepNet takes raw bio-signals, such as EEG, EOG, and EMG as input, and transforms them into normalized spectrograms with signal processing. The normalized signals then pass through the epoch encoder, which extracts input-invariant features using bi-gated recurrent unit blocks and the attention mechanism. With this approach, the authors reported the robust performance of the model across various unseen sleep datasets in experiments using multi-channel inputs.

U-sleep (Perslev et al., 2021) improved their previous work, U-time (Perslev et al., 2019). This was designed to exhibit higher generalization performance than U-time. Compared with U-time, U-sleep has deeper layers and uses simpler cross-entropy loss rather than the dice loss used in U-time. U-sleep takes two input signals (EEG and EOG) for any arbitrary position and returns the segmented sleep stages. The authors demonstrated the generalized performance of U-sleep through experiments using various sleep cohort datasets.

CoSleepNet (Efe and Ozsen, 2023) utilized only one EEG channel and one EOG channel to increase practicality by utilizing as few channels as possible while utilizing multiple channels. Specifically, the authors proposed a hybrid neural network architecture using focal loss and discrete cosine transform methods. Validation on the Sleep-EDF dataset resulted in the highest score being 87.1% accuracy, 81.8% kappa value, and 79.8% F1-score in overall performance.

The use of multi-channel signals, rather than single-channel signals, demonstrated higher performance for sleep stage scoring. Nevertheless, improving the classification performance using fewer channels is crucial for practicality.

## 3 SeriesSleepNet

**FIGURE 1:**
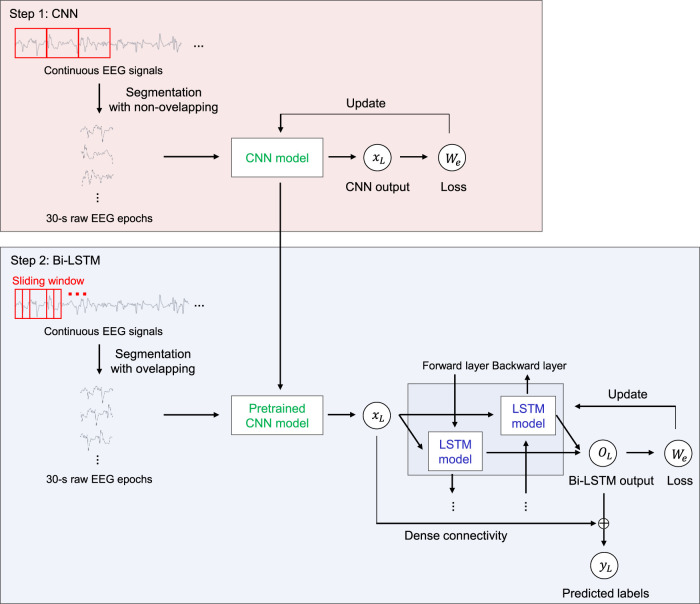
An overview of the SeriesSleepNet architecture. The proposed framework consists of two steps. In step 1, 30-s raw EEG signals are used as inputs of CNN for extracting time information in a single sleep epoch (inter-epoch). The pre-trained CNN model was trained to be used in the next step while learning temporal information in the inter-epoch. In step 2, the bi-LSTM uses the serial information (intra-epoch) extracted from the above CNN as input. Here, the pre-trained CNN used for training bi-LSTM takes sequences of 30-s raw EEG signals, which is augmented by the sliding window method. Subsequently, the input and output features of the bi-LSTM are combined with the inner product to classify the sleep stages. The red square represents 30-s EEG epochs in both steps. The *fs*, *x*_*n*_, *o*_*n*_, *W*_*n*_, *y*_*n*_, *L*_*n*_ and ⊗ denote the sampling rate of the input signal, output features of CNN, output features of bi-LSTM, the weight of adaptive loss function, predicted label, and a calculated loss of *n*th epoch, and operation symbol “inner product,” respectively.

We propose *SeriesSleepNet*, a novel automatic sleep stage classification model composed of a CNN and bi-LSTM with residual fusion (Figure 1). The entire learning process involves two-stage training. In the first stage, the CNN module is pre-trained. Raw EEG signals of a single sleep epoch are used as input for training the CNN module, and the module learns the temporal information of one sleep epoch (intra-epoch). The next stage is for training the bi-LSTM module. A pre-trained CNN is used in this stage. The pre-trained CNN takes input signals that are partially augmented using the sliding window method and returns the output features. The resulting output features are used as the input for the bi-LSTM. After the bi-LSTM returns the output feature, the input and output features of the bi-LSTM are combined with the inner product, and the combined features are used to classify the sleep stages for each epoch (inter-epoch). Consequently, the sequential information of the adjacent epoch can be considered for sleep stage scoring. We also proposed an adaptive cross-entropy loss function that provides additional weights adaptive to the F1-scores of the sleep stages from the previous training epoch and used them as both CNN and bi-LSTM.

### 3.1 CNN for training intra-epoch

Consecutive EEG signals that comprise 30-s interval PSG epochs are exploited herein. In the first layer, we applied a kernel size of *fs* (sampling rate) × 4 to extract the lowest frequency band that we want to extract, 0.5 Hz. Subsequently, layers with a kernel size of *fs*/12 and 3 were applied to consider both the frequency and temporal information of EEG signals (Supratak et al., 2017). To reduce the computational cost of extracting depth-wise features between learned feature maps, we also applied depth-wise convolution layers with a kernel size of one. The details of the CNN architecture are presented in Table 2. After each convolutional layer, the rectified linear unit is selected as the activation function (*i*.*e*., *relu*(*x*) = max(0, *x*)). The output of the CNN is as follows.

$$x_{i}=CNN\left(eeg_{i}^{\mathrm{raw}}\right)$$ (1)

where $eeg_{i}^{\mathrm{raw}}$ is a 30-s raw EEG signal of the *i*th sleep epoch and *x*_*i*_ is the output features. We also used the Adam optimizer (Kingma and Ba, 2014) to boost the learning process for our model, and the hyperparameters were selected as a batch size of 128, a learning rate of 1e-3, and a weight decay of 1e-4. The output features were used as the input for the bi-LSTM.

**TABLE 2:** Details of layers and hyperparameters of CNN in the SeriesSleepNet.

Number	Layers	Number of filters	Kernel size (H × W)	Stride (H × W)	Activation function
1	2D Convolution layer 1	16	1 × (*fs* × 4)	1 × (*fs*/16)	ReLU
2	2D Convolution layer 2	32	1 × (*fs*/12)	1 × 1	ReLU
3	2D Convolution layer 3	64	1 × 3	1 × 1	ReLU
4	Depth-wise 2D Convolution layer	16	1 × 1	1 × 1	—
5	2D Batch normalization	16	—	—	ReLU
6	Dropout (0.5)	—	—	—	—
7	Flatten	—	—	—	—
8	Fully-connected layer 1 (1024)	—	—	—	ReLU
9	Fully-connected layer 2 (256)	—	—	—	ReLU
10	Dropout (0.5)	—	—	—	—
11	Classification layer (number of classes)	—	—	—	Softmax

### 3.2 Bi-LSTM for training inter-epoch with partial data augmentation

As shown in Table 3, the size of the input features is 256, which is equal to the size of the output features of the CNN. LSTM cells, based on the sequence length, are located in the forward and backward directions. The sliding window method was used for partial data augmentation of the input signals in this training stage. This augmentation is for the input data to contain the intermediate feature between two sleep epochs. As the sleep stage is scored with 30-s intervals, the important features for scoring the sleep stage could be placed across two sleep epochs (for example, the feature could start from 28-s segments of a sleep epoch and last until 2-s segments of the next sleep epoch). For considering this, we utilized 4 s of overlapping segmentation with steps of 1 s using the sliding window method to learn the connected time information. As a result, 4 series of augmented data were used as inputs, and the model could consider the 4-s intermediate feature between the current and next sleep epochs. The augmented input signals pass through the pre-trained CNN, and the output features of the CNN are used as the input for the bi-LSTM. The size of all the bidirectional hidden states is 512 (256 × 2). These cells calculate the weights using the features received from the previous LSTM cell and the corresponding output features of the CNN. They learn the sequential information between adjacent epochs.

**TABLE 3:** Details of hyperparameters of bi-LSTM in the SeriesSleepNet.

Hyperparameters	Value
Learning rate	1e-4
Batch size	64
Weight decay	1e-5
Length of sequence	{10, 20, 30, 40}
Size of hidden state	256
Size of input features	256
Number of RNN layers	2
Dropout rate	0.1

The output features containing the sequential characteristics and input features containing the temporal information of each sleep stage are combined with the inner product to classify the sleep stage. This technique aims to connect and combine features from a CNN into features from an LSTM in a feature-wise manner. Each element of the resulting feature can contain feature information from the output of the CNN. In contrast, DeepSleepNet (Supratak et al., 2017) uses the concept of skip connection using the sum of the input features and the output features as the input features of the next layer, such as the skip connection of ResNet (He et al., 2016); however, it may blur the information from the input features (Huang et al., 2017). Therefore, the flow of information is in this manner. In this regard, we used a connection method based on feature fusion using an inner product (Hu et al., 2017; Mai et al., 2020). This has the effect of estimating the similarity between two features (Mai et al., 2020); thus, the resulting feature considers both the input and output features of bi-LSTM according to their importance in classification.

### 3.3 Adaptive cross-entropy loss

The cross-entropy loss function (Rubinstein and Kroese, 2013) is widely used and is known to be highly efficient in deep learning. However, because of the different portions of sleep stages, a class imbalance problem occurs, and sometimes this loss function can be limited. Specifically, the N1 stage is often misclassified because the proportion of the N1 stage is very low during sleep (Chambon et al., 2018). To address this problem, AttnSleep (Eldele et al., 2021) and SleepFCN (Goshtasbi et al., 2022) applied a fixed class-wise loss weight based on the rarity of classes. Rarity-based weighting methods are a natural and intuitive approach, but it is based on heuristics. This kind of heuristic approach is influenced by inductive bias. While inductive bias would be helpful in some cases but may work as an inhibitor of effective training when the properties of the dataset are unusual. In this respect, to reduce the inductive bias of the fixed weighting methods, we propose a data-driven class-wise weighting method for these cross-entropy functions. This process is applied to calculating the F1-score of each class using the training set and to adjust the weight according to the resulting F1-score when every training epoch is completed. In summary, we used the normal unweighted cross-entropy loss function for ten training epochs to warm up the CNN and bi-LSTM.

The proposed loss function is expressed as follows:

$$loss=\left\{\begin{array}{cc} -\underset{i}{\sum }\left[p_{i}\log{\hat{p}}_{i}\right]\; & \text{if }e\leq \frac{e_{\max}}{3},\\ -\underset{i}{\sum }\left[W_{i}p_{i}\log{\hat{p}}_{i}\right]\; & \text{otherwise.} \end{array}\right.$$ (2)

$$\;\;W_{i}={\left(1-\log_{K}CF_{i}\right)}^{\gamma }$$ (3)

where *p*_*i*_ and ${\hat{p}}_{i}$ denote the probability of true label *i* and the posterior probability of predicted label *i*, respectively. *e* and *e*_max_ indicate the current training epoch count and the max training epoch value, respectively. *K* and *γ* are hyperparameters to adjust the degree of weight factor *W*_*i*_, and *CF*_*i*_ denotes the class-wise F1-score of class *i* resulting from the last training epoch. We conducted a grid search to determine the proper values of *K* and *γ* for this study and the values were selected as ten and three respectively (Supplementary Table S1). To prevent gradient explosion caused by a small value of *CF*_*i*_, we adjusted the value to 1e-4 when smaller than 1e-4. With the adaptive loss function, we hypothesized that the model could update its parameters by considering the difficulties of each class, avoiding overfitting the parameters to classes that are easy to learn.

We also applied label smoothing (Müller et al., 2019) to both the original cross-entropy and adaptive cross-entropy loss. Label smoothing converts hard labels into soft labels by using the following equation:

$$y_{k}^{LS}=y_{k}\left(1-\alpha \right)+\alpha /K$$ (4)

where $y_{k}^{LS}$ is the smoothed label of class *k*, *y*_*k*_ is the hard label of class *k*, *α* is a smoothing parameter, and *K* is the number of classes. In this work, we set the parameter *α* as 0.1 for both the original cross-entropy and adaptive cross-entropy.

## 4 Model training algorithm

We configured the parameters of the bi-LSTM after learning all the CNN parameters. First, CNN learns the temporal features of each sleep stage with one EEG epoch. Subsequently, the bi-LSTM learns the sequential information of adjacent epochs using the output features from the CNN. At this time, the output of the CNN already used as the input of the bi-LSTM is combined with the output of the bi-LSTM by the inner product. We set the maximum training epoch *e*_max_ to 30 for training both the CNN and bi-LSTM.

### 4.1 Step 1: training the CNN

Raw EEG signals from a single sleep epoch are used for the CNN. When the training epoch count *e* is lower than 11, the model uses the conventional cross-entropy loss function for warm-up; if *e* is higher than 11 or equal to 11, the adaptive is used cross-entropy loss function. As we evaluated the model with a nested cross-validation method, we separated the training set into 90:10 ratios and used a smaller portion as the validation set. The model was validated for every training epoch using the validation set, and the model parameter recorded with the highest evaluation score in the validation set was saved and used for testing. To use the proposed adaptive cross-entropy loss function, we obtained the F1-score for each class of the training set for each training epoch.

### 4.2 Step 2: training the Bi-LSTM

The pre-trained CNN module is transferred into the bi-LSTM training stage. The CNN module takes the partially augmented EEG signals as input and returns the output features, which are used as inputs for bi-LSTM. Note that we do not use the output features of the bi-LSTM directly for the final classification, but the features after combining with the input of the bi-LSTM using the inner product. In other words, the output of the CNN (1 × 256) is multiplied by the transposed output of the LSTM (512 × 1), resulting in a combined feature (512 × 256). The combined feature passes through the rectified linear unit (ReLU) activation and is pooled with average pooling. The 512 × 1 feature was mapped back to 1 × 5 through two fully connected layers, and the probability corresponding to each sleep stage was then calculated using the softmax activation.

Similar to the process for the pretraining CNN module, The conventional cross-entropy loss function is used if the training epoch count *e* is lower than 11, and the proposed adaptive cross-entropy loss function is used if *e* is greater than or equal to 11. The F1-score of each class in the training set was calculated for each training epoch and was used to update the weight of the modified loss function. The LSTM model parameter was also saved if it recorded the highest evaluation score in the validation set and was used for testing.

## 5 Experiments

### 5.1 Public datasets

Two datasets are used in this study. The Sleep Cassette (SC) subset from the Sleep-EDF (Goldberger et al., 2000; Kemp et al., 2000), SHHS datasets (Quan et al., 1997) are shown in Table 4. This study is mainly focused on the validation of healthy, normal sleep recordings.

**TABLE 4:** Details of two public datasets used in our experiments.

Dataset	Num. of subjects	EEG channel	Sampling rate (Hz)	Wake	N1	N2	N3	REM	Total epochs
Sleep-EDF	20	Fpz-Cz	100	8,285	2,804	17,799	5,703	7,717	42,308
				19.6%	6.6%	42.1%	13.5%	18.2%	
SHHS	329	C4-A1	125	46,319	10,304	142,125	60,153	65,953	324,854
				14.2%	3.2%	43.8%	18.5%	20.3%	

#### 5.1.1 Sleep-EDF

This dataset comprised 20 healthy subjects aged 25–34. The PSG recordings included two EEG channels (Fpz-Cz and Pz-Oz), one horizontal EOG channel, and one submental chin EMG channel. The EEG and EOG signals had a sampling rate of 100 Hz and the EMG signals were sampled at 1 Hz. These data were labeled by sleep experts into one of eight classes (“W,” “R,” “1,” “2,” “3,” “4,” “M,” and “?”) according to R&K standards. Sleep stage “M” indicates movement time and “?” denotes not scored. We merged “3” and “4” into “3,” which is the same sleep stage according to the AASM standard (Sinton and McCarley, 2004). Consequently, we classified the five sleep stages according to with the AASM standard. Previous studies have reported that classification performance using the Fpz-Cz EEG channel was higher than that using the Pz-Oz EEG channel (Hsu et al., 2013; Supratak et al., 2017). Therefore, we used only the Fpz-Cz EEG channel in the SC subset. Moreover, we only exploited the recordings before and after 30 min of sleep periods (*i.e.*, the in-bed parts from lights off time to lights on time) (Phan et al., 2018).

#### 5.1.2 SHHS

This consisted of two rounds of PSG recordings. We used only the first round (SHHS-1) because all the data had the same sampling data. This study included 5,805 subjects aged 40 years and older and an apnea hypopnea index (AHI) to score apnea levels. PSG recordings included two bipolar EEG channels (C4-A1 and C3-A2), two EOG channels (right and left), one EMG channel, and one ECG channel. The EEG, EOG, and EMG channels had a sampling rate of 125 Hz, whereas the EOG channels had a sampling rate of 50 Hz. Each of the 30-s epochs is labeled R&K standard by an expert, and we reorganized S3 and S4 stages into the N3 stage by AASM, eventually converting to five sleep stage classifications: Wake, REM, N1, N2, and N3. In this work, we used the C4-A1 EEG channel (Sors et al., 2018) and the data quality was outstanding for more than 6 h. We included data with no apnea (AHI < 5) to classify healthy subjects. We attempted to exclude as many other factors as possible because sleep apnea itself is a variable that greatly affects EEG signals, following the experimental setups of AttnSleep (Eldele et al., 2021). Consequently, 329 subjects were selected to build the SHHS dataset.

### 5.2 Experimental setup

For the Sleep-EDF dataset, we evaluated SeriesSleepNet using leave-one subject-out (LOSO) nested cross-validation. This method is an inter-target classification approach in which a generalized network is learned using data from the subject pool (training stage), and the learned knowledge is transferred to a new subject (test stage) (Fahimi et al., 2019). In this respect, this method is effective in evaluating the generalization ability of the model compared to the existing method of dividing the training and test sets (Lee et al., 2022). Therefore, we used 20 folds cross-validation in the Sleep-EDF dataset (Phan et al., 2018). For the SHHS dataset, we conducted 20 folds cross-validation in a nested manner by dividing the data into 20 groups, as in AttnSleep (Eldele et al., 2021). The process was repeated five times, as in previous studies (Shu et al., 2019; Rahman et al., 2020), and the mean and standard deviation were calculated.

To validate our model, we compared SeriesSleepNet to several state-of-the-art methods. For a fair comparison, we only used models that performed under the same experimental setups as ours (single channel EEG as input, LOSO cross-validation for Sleep-EDF, and 20 folds cross-validation for SHHS) with the same number of total sleep epochs in our study (Sleep-EDF = 42,308, SHHS = 324,854).

Note that we utilized and implemented the model of Olesen *et al.* (Olesen et al., 2021) with a single-channel EEG input (Fpz-Cz for Sleep-EDF; C4-A1 for SHHS) without signal preprocessing for a fair comparison to other models and adjusted the learning rate from 0.1 to 0.01 because the model couldn’t train its parameters with the original one in our experimental setups. We also implemented RobustSleepNet (Guillot and Thorey, 2021) and U-sleep (Perslev et al., 2021) and tested them in our experimental setups for a fair comparison because they were originally designed to use multi-channel input, utilized with different data samples, and evaluated with different methods.

The overall F1-score, accuracy, and kappa value were used as performance measurement criteria. These performance metrics are indicators of commonly used model performance (Suk et al., 2014; Chambon et al., 2018). In particular, the F1-score is often used as an indicator when the data label is unbalanced (Phan et al., 2019). In this regard, we measured the class-wise F1-score.

### 5.3 Implementation

The proposed method and baseline models were implemented in Python 3.7 using *Pytorch v1.3.1*. For training and testing, a computer in Intel Core i9-10980 KE, 3.00-GHz CPU and 128-GB RAM, and an NVIDIA GTX 3090 GPU was used.

## 6 Results and discussion

### 6.1 Classification performance of sleep stage

#### 6.1.1 Sleep-EDF

**FIGURE 2:**
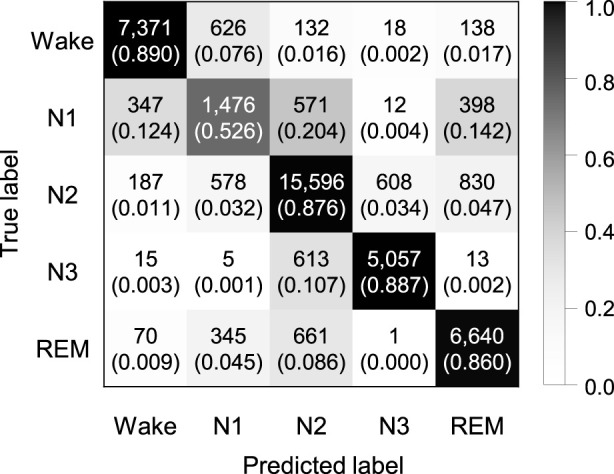
Confusion matrix of 5-class sleep stage classification with the sequence length in bi-LSTM of 30 using the Sleep-EDF dataset. A total of 42,308 epochs were used, and the values in the parenthesis indicate the normalized values.

Experiments were performed to explore whether the sequence length is an important factor in our proposed model. We applied by applying sequence lengths of 10, 20, 30, and 40 in bi-LSTM, and compared the classification performance for sleep stage scoring. Figure 2 shows the confusion matrix of SeriesSleepNet according to the sequence length of 30 in bi-LSTM from the Sleep-EDF dataset. As shown in Supplementary Figure S1, the overall F1-scores were 78.9%, 79.5%, 79.8%, and 79.6% for the sequence lengths of 10, 20, 30, and 40, respectively. Moreover, we achieved overall accuracies of 84.1%, 84.6%, 84.8%, and 84.1% and kappa values of 0.783, 0.789, 0.792, and 0.790, respectively. In the F1-score in the N1 stage, the classification performance using a sequence length of 30 was higher than that using sequence lengths of 10, 20, and 40. More specifically, when the sequence length was 30, the F1-score of the N1 stage was 49.4%, which was higher than the 47.2%, 48.8%, and 49.1% at 10, 20, and 40, respectively.

**FIGURE 3:**
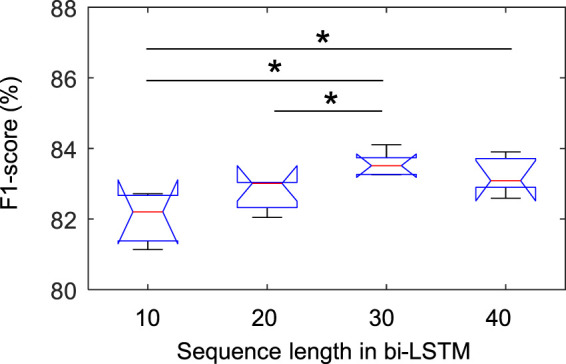
Comparison of class-wise F1-score in REM stage according to sequence length in bi-LSTM using the Sleep-EDF dataset. * indicated a significant difference with Fisher’s least significant difference procedure for multiple comparisons (*p* < 0.05).

**FIGURE 4:**
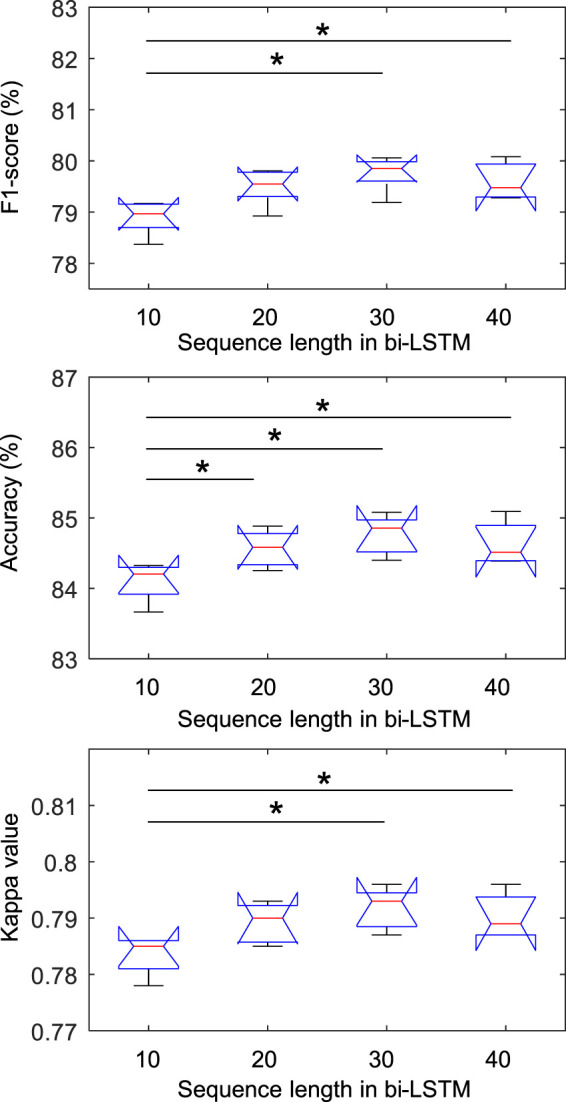
Comparison of overall metrics according to sequence length in bi-LSTM using the Sleep-EDF dataset. * indicated a significant difference with Fisher’s least significant difference procedure for multiple comparisons (*p* < 0.05).

Figure 3 shows the statistical results of the performance according to the sequence length. In the class-wise F1-score, there was a statistical difference only in the REM stage (chi-square = 0.007), and there was no difference in other stages. In particular, in the REM stage, when the sequence length was 30, the F1-score was significantly higher than when it was 10 and 20, which was applied to Fisher’s least significant difference procedure for multiple comparisons. As shown in Figure 4, the overall metrics also had a higher performance at 30 than when the sequence length was 10 or 20 (F1-score: Chi-square = 0.017, Accuracy: Chi-square = 0.013, Kappa value: Chi-square = 0.016). There was no statistical difference in performance when sequence lengths of 30 and 40 were used, but a longer sequence length increased the calculation time, so we applied a sequence length of 30 to proceed with future analysis.

Although most recent sleep stage scoring models have been developed and tested using datasets labeled with the AASM criterion, the R&K criterion is still widely used for sleep stage scoring (Malekzadeh et al., 2022). To verify the general performance of the model when it was used for practical applications, we conducted an additional experiment on the proposed model with the Sleep-EDF dataset labeled with the R&K criterion. Table 5 lists the overall performance for each criterion. The overall performance of the proposed model decreased when we experimented with the R&K criterion compared with the AASM criterion. Specifically, the overall F1-score decreased from 79.8% to 74.2%, and the accuracy and kappa decreased from 84.8% to 82.2% and 0.792 to 0.760, respectively. Nevertheless, there was no statistical difference between the F1-score (*p* = 0.081) and accuracy (*p* = 0.078). On the other hand, there was a statistical difference in the kappa value (*p* < 0.001), which was higher when AASM was applied than when R&K was applied, but this is considered a natural phenomenon because the chance level decreases as the number of classes increases. Supplementary Figure S2 is shown in the confusion matrix applied to R&K using the Sleep-EDF dataset.

**TABLE 5:** Comparison of the classification performance by sleep stage scoring criteria using the Sleep-EDF dataset.

Criteria	Overall metrics		
	F1-score (%)	Accuracy (%)	Kappa
AASM (5-class)	79.8 ± 0.3	84.8 ± 0.3	0.792 ± 0.004
R&K (6-class)	74.2 ± 1.5	82.2 ± 0.1	0.760 ± 0.001

Mean ± standard deviation.

#### 6.1.2 SHHS

**FIGURE 5:**
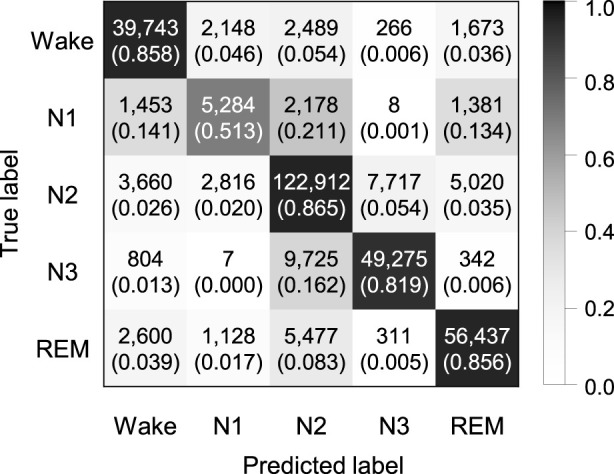
Confusion matrix of 5-class sleep stage classification with the sequence length in bi-LSTM of 30 using the SHHS dataset. A total of 324,854 epochs were used, and the values in the parenthesis indicate the normalized values.

Figure 5 shows the confusion matrix of SeriesSleepNet according to a sequence length 30 of in bi-LSTM from the SHHS dataset. The classification performance resulted in an overall F1-score of 77.7%, overall accuracy of 84.1%, and a kappa value of 0.777. The class-wise F1-score was 83.8%, 48.6%, 86.0%, 83.6%, and 86.4% for Wake, N1, N2, N3, and REM stages, respectively. The result with the SHHS dataset showed a slightly lower performance compared to the result with the Sleep-EDF dataset, except for the class-wise F1-score of the REM stage.

### 6.2 Ablation study

We performed ablation experiments using the Sleep-EDF dataset to prove the effectiveness of each component. The proposed framework consists of four components: i) basic architecture based on CNN with bi-LSTM, ii) residual fusion when the outputs of CNN and bi-LSTM are combined in the final output, iii) partial data augmentation in the input of bi-LSTM, and iv) the proposed adaptive cross-entropy loss function. We calculated the sleep stage classification performance with and without each component, as listed in Table 6. As a result of the Kruskal-Wallis test, there were statistical differences in all overall metrics and class-wise F1-scores (*p* < 0.001). Supplementary Table S2 shows the detailed description of each pairwise comparison between the two rows of Table 6. Here, the bi-LSTM baseline indicates that the output of the CNN was not combined with the output of the LSTM; only the bi-LSTM output was used to predict the sleep stages.

**TABLE 6:** Classification performance of ablation study using Sleep-EDF dataset.

CNN	bi-LSTM				Loss function		Overall metrices			Class-wise F1-score(%)				
	Baseline	SC	RF	PDA	CE	ACE	F1-score (%)	Accuracy (%)	Kappa	Wake	N1	N2	N3	REM
◦	×	×	×	×	◦	×	71.0 ± 0.5	79.7 ± 0.3	0.721 ± 0.004	85.1 ± 0.3	26.7 ± 2.1	85.0 ± 0.5	86.4 ± 0.9	72.0 ± 0.4
◦	×	×	×	×	×	◦	72.2 ± 0.5	79.4 ± 0.5	0.719 ± 0.006	85.1 ± 0.6	32.9 ± 1.0	85.2 ± 0.4	86.3 ± 0.4	71.7 ± 1.0
◦	◦	×	×	×	◦	×	76.9 ± 0.4	83.5 ± 0.3	0.773 ± 0.004	88.2 ± 0.8	40.9 ± 1.7	87.2 ± 0.2	87.7 ± 0.2	80.6 ± 0.4
◦	◦	×	×	×	×	◦	78.1 ± 0.3	84.4 ± 0.2	0.786 ± 0.003	89.6 ± 0.9	42.7 ± 1.0	87.8 ± 0.2	88.2 ± 0.1	82.1 ± 0.5
◦	◦	◦	×	×	◦	×	76.9 ± 0.4	83.4 ± 0.2	0.772 ± 0.003	87.9 ± 0.2	41.0 ± 1.5	87.8 ± 0.2	87.8 ± 0.2	80.5 ± 0.6
◦	◦	◦	×	×	×	◦	78.2 ± 0.4	84.2 ± 0.3	0.783 ± 0.004	88.9 ± 0.7	44.1 ± 0.8	87.8 ± 0.2	88.4 ± 0.2	81.8 ± 0.7
◦	◦	×	◦	×	◦	×	77.1 ± 0.4	83.5 ± 0.2	0.773 ± 0.003	88.4 ± 0.9	41.5 ± 1.3	87.2 ± 0.2	87.7 ± 0.2	80.5 ± 0.9
◦	◦	×	◦	×	×	◦	78.5 ± 0.1	84.5 ± 0.1	0.786 ± 0.002	89.6 ± 0.7	44.4 ± 0.8	87.8 ± 0.2	88.3 ± 0.2	82.1 ± 0.5
◦	◦	×	×	◦	◦	×	78.4 ± 0.5	84.3 ± 0.3	0.784 ± 0.004	89.7 ± 1.2	44.8 ± 2.0	87.3 ± 0.2	88.0 ± 0.2	82.0 ± 0.7
◦	◦	×	×	◦	×	◦	79.5 ± 0.4	84.7 ± 0.3	0.791 ± 0.004	**90.1** **±** **0.9**	48.1 ± 0.9	87.9 ± 0.2	88.3 ± 0.1	82.8 ± 0.5
◦	◦	◦	×	◦	◦	×	78.9 ± 0.3	84.5 ± 0.2	0.787 ± 0.003	89.2 ± 0.6	45.4 ± 0.9	87.6 ± 0.3	88.1 ± 0.3	82.7 ± 0.5
◦	◦	◦	×	◦	×	◦	79.2 ± 0.3	84.6 ± 0.3	0.789 ± 0.004	89.4 ± 0.6	47.3 ± 0.6	**88.0** **±** **0.2**	**88.5** **±** **0.2**	82.7 ± 0.8
◦	◦	×	◦	◦	◦	×	78.8 ± 0.2	84.5 ± 0.2	0.788 ± 0.002	89.7 ± 1.1	45.9 ± 0.6	87.7 ± 0.3	88.0 ± 0.2	82.7 ± 0.9
◦	◦	×	◦	◦	×	◦	**79.8** **±** **0.3**	**84.8** **±** **0.3**	**0.792** **±** **0.004**	89.7 ± 0.8	**49.4** **±** **1.4**	87.9 ± 0.2	88.2 ± 0.2	**83.6** **±** **0.3**

◦ = activation; × = inactivation; Mean ± standard deviation; Skip-connection = SC; Residual fusion = RF; partial data augmentation = PDA; adaptive cross-entropy = ACE; original cross-entropy = CE.

Bold values indicate the highest score.

#### 6.2.1 Basic architecture

Using the CNN without bi-LSTM, F1-scores of 71.0% and 72.2% were achieved using the original cross-entropy and proposed adaptive cross-entropy, respectively. The role of a CNN is to extract temporal information within one epoch as an intra-epoch time series. In contrast, the use of bi-LSTM without residual fusion under the same conditions resulted in an F1-score performance improvement of between 4% and 6% compared to when only the CNN was used for the classification. The bi-LSTM module learns sequential information between adjacent epochs as an inter-epoch time series. In this regard, the sleep stage contains crucial connected information within or between EEG samples, which is thoroughly learned. Furthermore, models using basic CNN without bi-LSTM have a class-wise F1-score of the N1 stage that is relatively lower at 26.7% and 32.9% of the original cross-entropy and the proposed adaptive cross-entropy, respectively. This suggests that the classification of the N1 stage requires more inter-epoch information than intra-epoch information. In other words, the CNN effectively extracts information on the sleep epoch, but it lacks the capability to extract inter-epoch information. For DeepSleepNet (Supratak et al., 2017) with similar models, the authors used a similar CNN-LSTM architecture to address the weakness of the CNN with the sequential model. Similarly, in our framework, we used a sequential model (bi-LSTM) to alleviate the low performance in class-wise classification when only the basic CNN architecture was used.

#### 6.2.2 Residual fusion

The use of residual fusion under the same conditions resulted in a performance improvement of 1%–1.5%. Combining the input and output of the bi-LSTM with the inner product helped to learn important sequential information between adjacent epochs. It has already been reported that an approach that considers both the input and output features of a model has the following advantages: it alleviates the vanishing gradient problem, strengthens feature propagation, encourages feature reuse, and substantially reduces the number of parameters (He et al., 2016; Huang et al., 2017).

In addition, we compared the skip connection used in DeepSleepNet (Supratak et al., 2017) and residual fusion. As expected, when combining the input and output in the bi-LSTM, using residual fusion was slightly higher in performance than using a skip connection. Skip-connection is also used to solve the vanishing gradient problem, but residual fusion is considered more efficient than skip connection because it applies location weight to fuse CNN information (intra-epoch) with bi-LSTM information (inter-epoch) (Wang et al., 2020).

#### 6.2.3 Partial data augmentation

We utilized partial data augmentation on the input of a pre-trained CNN, used for training bi-LSTM, to increase the amount of data and extract the borderline information between sleep epochs. When partial data augmentation was not applied, the overall F1-score dropped by approximately 1.5%. The sliding window method is a data augmentation strategy used to apply EEG signals to deep learning (Lashgari et al., 2020). Even in our proposed framework, the sliding window is considered to contribute directly to improving the sleep stage classification performance by increasing the amount of data to be trained in the bi-LSTM.

#### 6.2.4 Adaptive cross-entropy loss function

Finally, an adaptive loss function based on cross-entropy was proposed. Under the same conditions, there were performance improvements in the overall metrics and the class-wise F1-score of the N1 stage, depending on the use of adaptive cross-entropy. The overall F1-score and class-wise F1-score of the N1 stage of the CNN standalone were 71.0% and 26.7%, respectively, not using the adaptive loss function. Using the proposed loss function in the experiments with CNN alone, the overall F1-score and class-wise F1-score of the N1 stage increased to 72.2% and 32.9%, respectively. When the proposed loss function was used with other components, it also exhibited increased performance. In the experiments with CNN and bi-LSTM, the overall F1-score and the performance of N1 stage increased from 76.9% to 78.1% and from 40.9% to 42.7%, respectively. Likewise, in other settings, the proposed loss function improved the overall F1-score and the class-wise F1-score of the N1 stage. These improvements reflect the alleviation of low performance in the N1 stage without using methods to reduce data imbalance problems, such as class-balanced re-sampling.

### 6.3 Effects of the adaptive cross-entropy loss function

To compare the efficacy of the adaptive cross-entropy loss function with other class-wise loss weighting methods, we conducted additional experiments using the loss functions used in AttnSleep (Eldele et al., 2021) and SleepFCN (Goshtasbi et al., 2022) with our framework (partial data augmentation and residual fusion). As shown in Supplementary Table S3, our loss weighting method showed the highest performance. There were statistical differences in all overall metrics and class-wise F1-scores through the Kruskal-Wallis test (*p* < 0.001). Detailed statistical results are also presented in Supplementary Table S4.

Even in another study (Tsinalis et al., 2016), using only the CNN model, the F1-score was 81% when a publicly available sleep dataset from 20 healthy young adults was used. However, this is believed to be possible because it solves the data imbalance problem using class-balanced random sampling within stochastic gradient descent optimization. In summary, the adaptive loss was able to resolve the low performance of the N1 stage, which is caused by the data imbalance problem by helping the model adaptively learn the imbalance between classes without knowing the entire distribution of data.

In particular, the class imbalance problem is important for sleep classification. Epochs in transitioning areas often contain characteristics of two or three sleep stages (Phan et al., 2019). In other words, the N1 stage is the transition area between the awake and sleeping state, in which brain activity changes the most. This reduces the performance of sleep stage classification. Consequently, increasing performance in the transitioning area is key to improving the performance of automatic sleep stage scoring. This is because it alleviates the error rate by adding weights to the frequently incorrect cases. In this regard, EEGNet (Lawhern et al., 2018), a deep learning framework known to be effective in brain-computer interfaces, also solves the class imbalance problem by assigning weights to the loss function.

Compared to other models, DeepSleepNet (Supratak et al., 2017) solves the class imbalance problem by oversampling the data. This means that a relatively low-class number of the other sleep stages, except for the N1 stage, is considered. However, our proposed loss function was effective for classifying the N1 stage, where the number of data classes was small compared to other sleep stages. We also used the F1-score for the weight, unlike AttnSleep (Eldele et al., 2021), which adjusts the weight in the loss function based on the distribution of each stage. This is believed to be far more effective to solve data imbalance, as it is designed to be more adaptive at the N1 stage, even when learning is poor.

**FIGURE 6:**
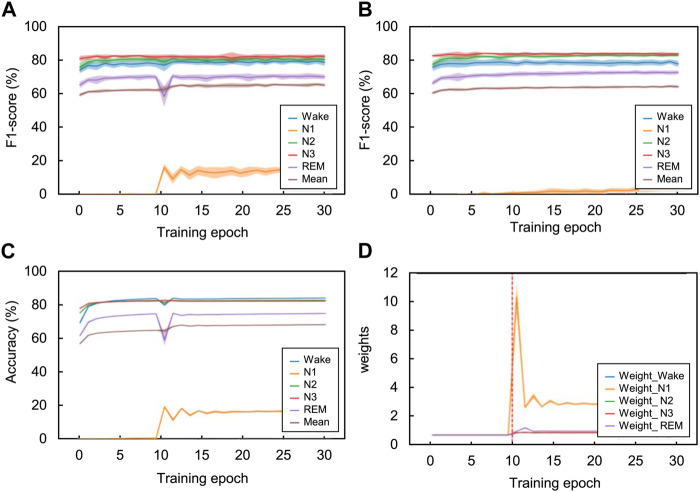
Comparison of the class-wise change in each sleep stage according to the training epoch using the SHHS dataset. Using the validation set, the class-wise F1-score changes according to the training epoch with **(A)** adaptive cross-entropy and **(B)** original cross-entropy. Additionally, using the training set with the adaptive cross-entropy, we investigated the changes in **(C)** class-wise F1-scores and **(D)** class-wise loss weight. Especially on the loss weight graph, the vertical red-dotted lines indicate the starting point using this loss function. The CNN model was used for training the temporal information. The value of the graphs are the mean results of 20 folds cross-validation and the shaded area indicates the standard deviation.

Figure 6 shows the effect of the adaptive cross-entropy loss function on training using the CNN model in the SHHS dataset. When the model was trained with the proposed loss function, the class-wise F1-score of the N1 stage in the validation set increased by approximately 15% compared to the model trained with the original cross-entropy loss function. As a result, the overall F1-score of the validation set during training also increased by approximately 5%. The weight of the proposed loss function adaptively changes according to changes in the class-wise F1-score of the training set. When the model was warmed with the original cross-entropy loss function (*i.e.*, training epoch from one to ten), the loss weights of each class were one. After the model was trained with the proposed loss function (*i.e.*, training epoch between 11 and 30), the loss weights of each class changed with adaption to the previous class-wise F1-score. Specifically, the loss weight of the N1 stage increased to approximately 11 when the previous F1-score of the N1 stage was near 0% and then decreased as the F1-score of the N1 stage increased. Likewise, when the class-wise F1-scores of the Wake, N2, and REM stages dropped due to the radical changes in training loss by using the proposed method, the proposed loss function adaptively changed the loss weights of the Wake, N2, and REM stages, increasing their F1-scores on the next training epoch.

In summary, each weight for classes is constantly changing, from one to two, according to their class-wise F1-scores for each training epoch. It might appear that weights converge certain values and do not change at all because of the large scale of *y* (weight) axis. However, the weights constantly adjust their values for fine-tuning. The performance of the Wake, N2, N3, and REM constantly changed while the mean performance increased after the 11th training epoch. The performance of the CNN model trained with original cross-entropy shows lower performance than adaptive cross-entropy, especially in N1 stage. Note that we adjusted the weight decay value from 1e-4 to 1e-5 when training the CNN model with original cross-entropy to get better baseline performance, because the model parameter wasn’t properly trained with the weight decay of 1e-4 and returned 0% of classification performance on N1 stage.

### 6.4 Comparison with state-of-the-art methods

Table 7 presents a comprehensive performance comparison between the proposed and other methods using single-channel EEG as input for 5-class classification (Wake, N1, N2, N3, and REM). We compared the classification performance using the same evaluation method for the sleep-EDF and SHHS datasets. Specifically, the sleep-EDF dataset was evaluated with 20 folds LOSO cross-validation, and the SHHS dataset was evaluated with 20 folds cross-validation. As a result, we presented the best performances of SeriesSleepNet, which can be achieved with the recommended hyperparameter setups, followed by (Fiorillo et al., 2021).

**TABLE 7:** Comparison of performance between SeriesSleepNet and state-of-the-art methods.

Dataset	Model	Year	Overall metrics			Class-wise F1-score(%)				
			F1-score(%)	Accuracy(%)	Kappa	Wake	N1	N2	N3	REM
Sleep-EDF	DeepSleepNet Supratak et al. (2017)	2017	76.6	81.9	0.76	86.7	45.5	85.1	83.3	82.6
	ResnetLSTM Sun et al. (2018)	2018	73.7	82.5	0.76	86.5	28.4	87.7	89.8	76.2
	Sors et al. (2018)	2018	76.0	81.8	0.75	86.0	44.0	86.1	85.5	78.3
	MultitaskSleepNet Phan et al. (2018)	2018	75.0	83.1	0.77	87.9	33.5	87.5	85.8	80.3
	SleepEEGNet Mousavi et al. (2019)	2019	76.6	81.5	0.75	89.4	44.4	84.7	84.6	79.6
	IITNet Seo et al. (2020)	2020	77.6	83.9	0.78	87.7	43.4	87.7	86.7	82.5
	SeqSleepNet+ Phan et al. (2020)	2020	78.4	85.2	0.80	90.5	45.4	88.1	86.4	81.8
	AttnSleep Eldele et al. (2021)	2021	78.1	84.4	0.79	89.7	42.6	88.8	90.2	79.0
	Olesen et al. (2021)	2021	69.3	76.8	0.68	78.3	30.2	84.7	83.6	69.7
	RobustSleepNet Guillot and Thorey (2021)	2021	75.3	82.7	0.77	86.7	36.6	86.0	86.4	81.0
	U-sleep Perslev et al. (2021)	2021	74.8	79.8	0.72	82.4	44.7	83.0	84.8	79.0
	XSleepNet2 Phan et al. (2021)	2021	**80.6**	86.3	**0.81**	92.2	**51.8**	88.0	86.8	83.9
	DeepSleepNet-Lite Fiorillo et al. (2021)	2021	78.0	84.0	0.78	87.1	44.4	87.9	88.2	82.4
	SleepFCN Goshtasbi et al. (2022)	2022	78.8	84.8	0.79	89.6	44.6	89.1	**90.6**	80.3
	LWSleepNet Yang et al. (2023)	2023	79.2	**86.6**	**0.81**	**92.4**	41.3	**90.2**	88.4	84.0
	SeriesSleepNet *(Ours)*		80.5	85.4	0.79	90.6	50.6	88.2	88.7	**84.4**
SHHS	DeepSleepNet Supratak et al. (2017)	2017	73.9	81.0	0.73	85.4	40.5	82.5	79.3	81.9
	ResnetLSTM Sun et al. (2018)	2018	69.4	83.3	0.76	85.1	9.4	86.3	87.0	79.1
	Sors et al. (2018)	2018	76.8	84.1	**0.78**	85.6	43.7	85.8	82.5	**86.3**
	MultitaskSleepNet Phan et al. (2018)	2018	71.2	81.4	0.74	82.2	25.7	83.9	83.3	81.1
	SleepEEGNet Mousavi et al. (2019)	2019	68.4	73.9	0.65	81.3	34.4	73.4	75.9	77.0
	AttnSleep Eldele et al. (2021)	2021	75.3	84.2	**0.78**	**86.7**	33.2	**87.1**	**87.1**	82.1
	Olesen et al. (2021)	2021	68.5	79.0	0.71	82.2	20.6	81.5	79.8	78.3
	RobustSleepNet Guillot and Thorey (2021)	2021	73.6	81.4	0.74	79.9	39.6	83.1	82.0	83.6
	U-sleep Perslev et al. (2021)	2021	76.9	84.1	**0.78**	85.5	43.6	85.8	83.3	86.0
	SeriesSleepNet *(Ours)*		**77.8**	**84.2**	**0.78**	84.0	**48.7**	86.3	83.7	**86.3**

Bold values indicate the highest score.

Using the Sleep-EDF dataset, our proposed SeriesSleepNet outperformed most other models in overall metrics. Specifically, only overall F1-score was lower than XSleepNet2 (Phan et al., 2021) at 0.1%. It is also 1.1% lower in overall accuracy than LWSleepNet (Yang et al., 2023). Finally, the overall Kappa values of the proposed model were 0.02 lower than XSleepNet2 (Phan et al., 2021) and LWSleepNet (Yang et al., 2023). On the other hand, in most performance metrics, the performance is similar to other models, and in particular, our model has the highest performance in the F1-score of the REM stage. The REM stage is associated with dreams with low wakefulness but high awareness in consciousness (Lee et al., 2022). So, it is very meaningful that the REM stage is highly accurate in that sleep-related diseases appear at this stage.

Using the SHHS dataset, SeriesSleepNet achieved higher performance in overall accuracy and F1-score. Moreover, SeriesSleepNet showed remarkably higher F1-score of N1 stages rather than baseline methods. AttnSleep (Eldele et al., 2021) achieved the highest F1-score of the Wake, N2, and N3 stages. The highest F1-score of the REM stage was achieved by SeriesSleepNet and the model of Sors et al. (2018). Higher overall kappa values were observed using SeriesSleepNet, U-sleep, AttnSleep, and the model of Sors *et al.* Above all, it is worth noting that the performance of the SHHS dataset, which is a large DB, is higher than that of state-of-the-art models.

In particular, our model performs better in the N1 stage than the other models but not in the N3 stage. This can be interpreted in two ways. The first is the adaptive cross-entropy loss function in the proposed model. The N3 stage account for a larger portion of the sleep stage than the N1 stage. In fact, the N1 stage is 6.6% in the Sleep-EDF dataset and 3.2% in the SHHS dataset, while the N3 stage exceeds 13% in both datasets. In addition, the performance for the N3 stage is relatively higher than that of the majority class. In this respect, the weight of the N3 stage could not benefit from the adaptive cross-entropy loss function, because it already shows sufficiently high performance compared to the N1 stage. The second is the difference in the model architecture itself between the proposed methods and other baselines. Other baseline models might be designed to show the advantage of more easily classifying the N3 stage than ours. For example, looking at the results of the ablation study, while the overall performance was the highest for the proposed model, the F1-score at the N3 stage was the highest for models without residual fusion in bi-LSTM.

Some papers present much worse performance than that mentioned in the original paper. This is because the experimental setups of the original paper and this study were different in terms of fair comparisons. In Sors et al. (2018), the overall performance of the model was not high enough compared to their original paper, with the F1-score, accuracy, and kappa values being reported as 78%, 87%, and 0.81, respectively. The original paper used the hold-out approach with a splitting ratio of 50% for training, 20% for validation, and 30% for testing, whereas we used 20 folds cross-validation. This method is effective in evaluating the generalizability of the model compared with the existing method of dividing the training and test sets (Lee et al., 2022). In this respect, 20 folds cross-validation has lower performance than other evaluation methods, even for the same model (Fahimi et al., 2019). In contrast, in Olesen et al. (2021), RobustSleepNet (Guillot and Thorey, 2021), and U-sleep (Perslev et al., 2021), the performance was not high enough compared to its original paper. Specifically, in the original study, PSG, including EEG, EOG, and EMG signals, was used as the input. However, we experimented only with single-channel EEG signals on each dataset. The evaluation methods used were also different. In this study, we evaluated the model with LOSO and 20 folds cross-validation in the Sleep-EDF and SHHS datasets, respectively. Under fair conditions, the performance of our model was better than that of the other models.

### 6.5 Limitation

The SeriesSleepNet has several limitations. First, as our model learns sequential information between epochs, it is necessary to explore all epochs of the sequence length. This can lead to delayed results when our model is applied in an online environment, such as sleep monitoring (Mikkelsen et al., 2019). Second, although SeriesSleepNet showed prominent classification performance among methods using single-channel signals, whether it maintains state-of-the-art performance compared with methods using multi-channel signals cannot be guaranteed. This is especially fatal when performance has a higher priority than the volume of the system, as in the medical field. Taking this into account, it is possible to expand SeriesSleepNet as a multi-channel-based method. Third, the performance of the proposed model depends on the learning level of the CNN. In other words, if the CNN does not train the sequential features of each sleep stage well, the subsequent bi-LSTM cannot guarantee high performance. Therefore, it is crucial to utilize appropriate methods that accurately extract the time features of each sleep stage. Fourth, the proposed adaptive loss function has hyperparameters such as *k* and *γ*; therefore, it is necessary to find the optimal value through experiments. If this is set incorrectly, a gradient explosion may occur. In our experiment, if a value less than 1e-4 was found in the confusion matrix, it was fixed to 1e-4 and used, but it would be beneficial to consider a method such as gradient clipping to supplement this. Lastly, additional experiments are required for further research. This study did not address subjects with insomnia. In this regard, it is necessary to verify whether it can be applied to the classification of sleep stages in patients with sleep disorders such as insomnia. Additionally, prospective studies using health and insomnia records are required. This is because, in terms of application, these are the main challenges in sleep classification. Furthermore, scoring arousals is also an important part of sleep scoring and classification. Therefore, this should be addressed in future studies.

## 7 Conclusion

We propose SeriesSleepNet, a novel automatic sleep stage classification model combined combines CNN and bi-LSTM, which is favorable for learning temporal information both intra-epoch and inter-epoch, that is effective with a time series model. Moreover, we applied the partial data augmentation based on the sliding window method to learn the connected time information in a small series. Finally, it contributed to the improvement of performance by proposing a loss function to solve the problem caused by sleep stage imbalance. As a result, the proposed framework performed better than state-of-the-art models in several evaluation metrics which were evaluated under the same experimental setups as ours. This automatic sleep stage scoring model could be used to diagnose sleep disorders or to measure sleep quality by evaluating sleep stages, which is believed to help reduce the burden on sleep professionals. In addition, the proposed framework would be applied not only to sleep but also to other time series fields such as weather forecasting, providing insight into the development of time series data-based deep learning models.

## Data Availability

The original contributions presented in the study are included in the article/Supplementary Material, further inquiries can be directed to the corresponding author.
