# Supplementary material for: SeriesSleepNet: an EEG time series model with partial data augmentation for automatic sleep stage scoring
Source: Front Physiol. 2023 Aug 28;14:1188678. doi: 10.3389/fphys.2023.1188678 (PMC10494443; doi:10.3389/fphys.2023.1188678)
Supplement: Supplementary file 1 [file DataSheet1.docx]

Supplementary Material

SeriesSleepNet: An EEG Time Series Model with Partial Data Augmentation for Automatic Sleep Stage Scoring

# Supplementary Figures and Tables

## Supplementary Figures


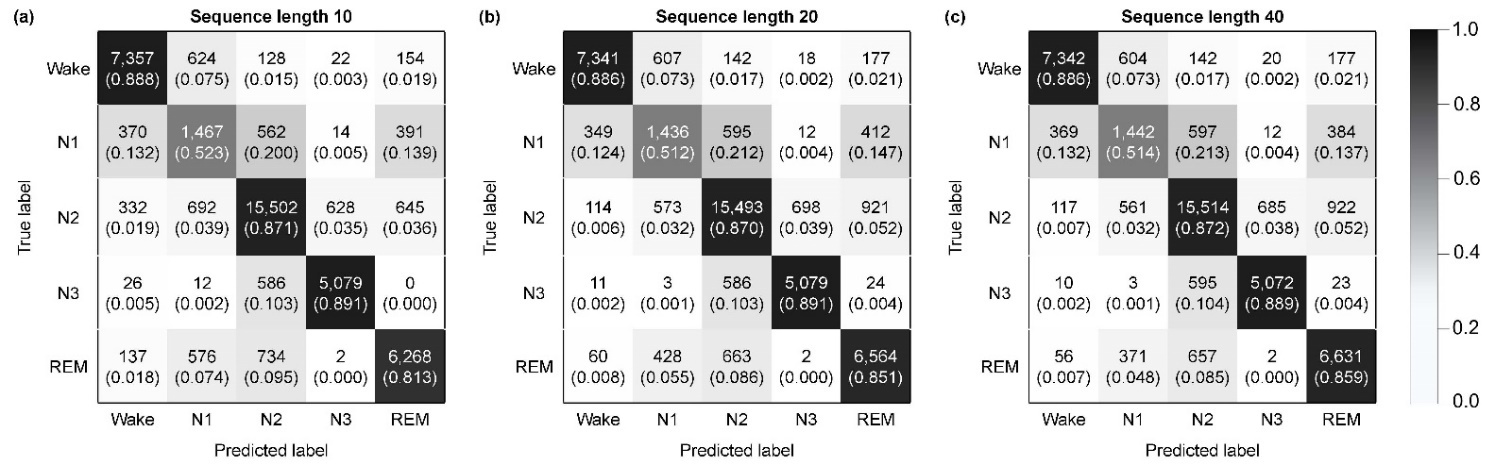


**Figure S1.** Confusion matrices of SeriesSleepNet with sequence length of **(A)** 10, **(B)** 20, and **(C)** 40 from representative trials in Sleep-EDF dataset. A total of 42,308 epochs were used in each matrix and the values within parenthesis represent the normalized values. (Wake: wakefulness, N1: non-REM 1, N2: non-REM 2, N3: non-REM 3, REM: rapid eye movement).


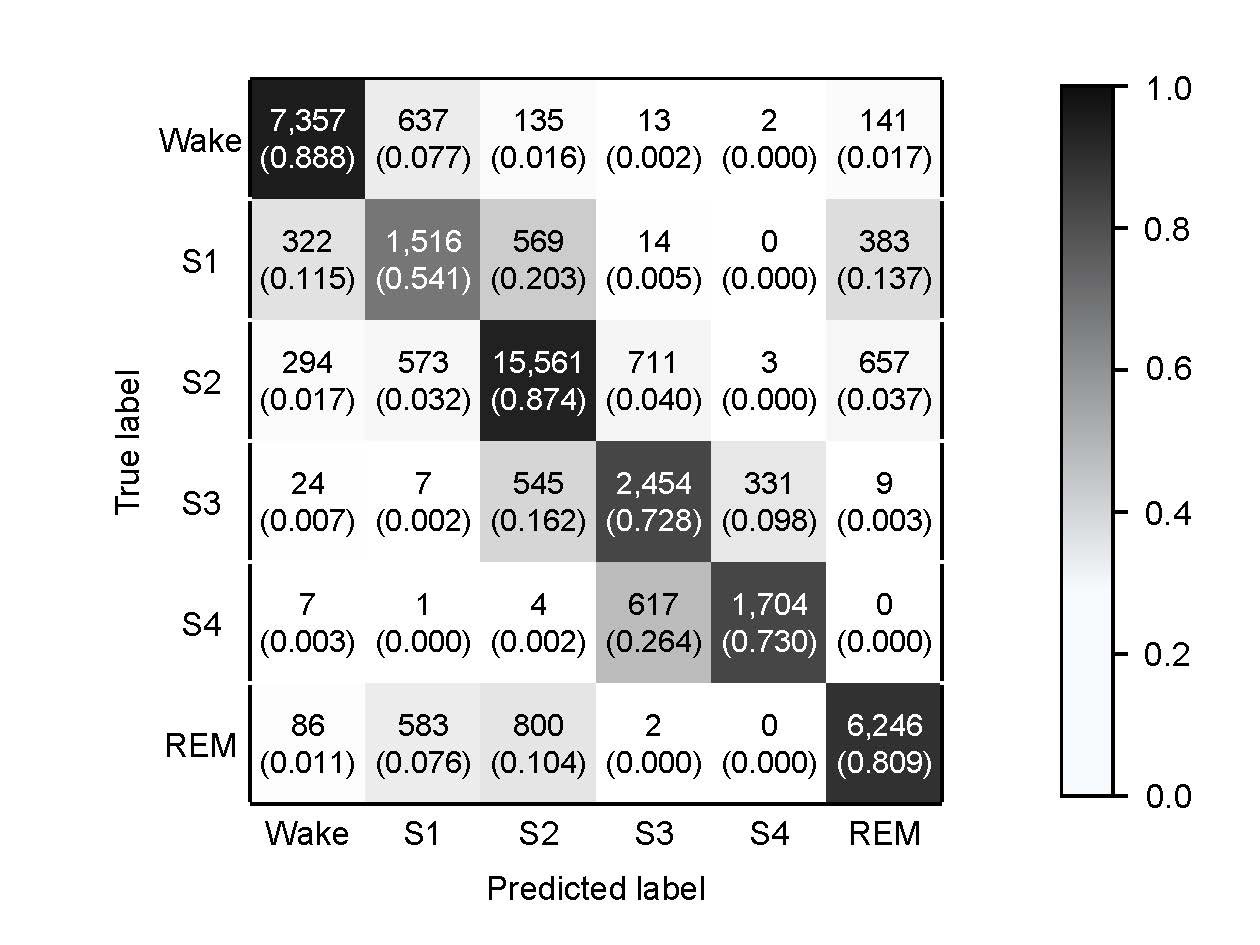


**Figure S2.** Confusion matrix of SeriesSleepNet in Sleep-EDF dataset labeled with R&K criterion from a representative trial. The values within parenthesis represent the normalized values. (Wake: wakefulness, N1: non-REM 1, N2: non-REM 2, N3: non-REM 3, REM: rapid eye movement).

## Supplementary Tables

**Table S1.** Results of grid search for the hyperparameters of loss function.

| **Macro**  **F1-score** | | **K** | | | |
| --- | --- | --- | --- | --- | --- |
|  |  | **2** | **5** | **10** | **100** |
| $\boldsymbol{\gamma}$ | **1** | 79.4 | 79.5 | 79.6 | 79.6 |
|  | **2** | 78.9 | 79.0 | 79.3 | 79.7 |
|  | **3** | 78.9 | 79.0 | **80.0** | 79.7 |
|  | **4** | 70.9 | 79.1 | 79.3 | 79.2 |

**Table S2.** Statistics results of ablation study.

| **Model Comparison** | **Overall metrics** | | | | **Class-wise F1-score(%)** | | | | |
| --- | --- | --- | --- | --- | --- | --- | --- | --- | --- |
|  | **F1-score(%)** | **Accuracy(%)** | **Kappa** | **Wake** | | **N1** | **N2** | **N3** | **REM** |
| Model 1 vs.  Model 14 | < 0.0001 | < 0.0001 | < 0.0001 | 0.0009 | | < 0.0001 | 0.0001 | 0.0021 | < 0.0001 |
| Model 2 vs.  Model 14 | < 0.0001 | < 0.0001 | < 0.0001 | 0.0010 | | < 0.0001 | 0.0001 | 0.0018 | < 0.0001 |
| Model 3 vs.  Model 14 | 0.0002 | 0.0012 | 0.0008 | 0.0502 | | 0.0002 | 0.0045 | 0.0467 | 0.0003 |
| Model 4 vs.  Model 14 | 0.0075 | 0.2315 | 0.1435 | 0.9504 | | 0.0021 | 0.4102 | 0.8157 | 0.0580 |
| Model 5 vs.  Model 14 | 0.0001 | 0.0009 | 0.0005 | 0.0182 | | 0.0002 | 0.0075 | 0.0903 | 0.0002 |
| Model 6 vs.  Model 14 | 0.0147 | 0.0667 | 0.0546 | 0.3675 | | 0.0167 | 0.5040 | 0.3276 | 0.0174 |
| Model 7 vs.  Model 14 | 0.0003 | 0.0012 | 0.0011 | 0.0995 | | 0.0004 | 0.0072 | 0.0502 | 0.0002 |
| Model 8 vs.  Model 14 | 0.0388 | 0.2907 | 0.1885 | 0.9257 | | 0.0243 | 0.7325 | 0.6300 | 0.0502 |
| Model 9 vs.  Model 14 | 0.0403 | 0.0933 | 0.0855 | 0.9876 | | 0.0521 | 0.0083 | 0.3353 | 0.0346 |
| Model 10 vs. Model 14 | 0.7678 | 0.7797 | 0.7794 | 0.5653 | | 0.7560 | 0.8278 | 0.4372 | 0.3593 |
| Model 11 vs. Model 14 | 0.0995 | 0.2502 | 0.2131 | 0.5973 | | 0.0964 | 0.1972 | 0.5549 | 0.2767 |
| Model 12 vs. Model 14 | 0.5445 | 0.5140 | 0.5439 | 0.7208 | | 0.5653 | 0.8765 | 0.1918 | 0.2138 |
| Model 13 vs. Model 14 | 0.2567 | 0.4747 | 0.4365 | 0.9876 | | 0.1866 | 0.2439 | 0.2502 | 0.2699 |

**Table S3.** Results of ablation study using other loss weighting method in Sleep-EDF dataset.

| **Weighting**  **methods** | **Overall metrics** | | | **Class-wise F1-score(%)** | | | | | |
| --- | --- | --- | --- | --- | --- | --- | --- | --- | --- |
|  | **F1-score(%)** | **Accuracy(%)** | **Kappa** | | **Wake** | **N1** | **N2** | **N3** | **REM** |
| **No weighting** | 78.1 $\pm$ 0.3 | 83.9 $\pm$ 0.2 | 0.779 $\pm$ 0.003 | | 89.0 $\pm$ 0.4 | 44.9 $\pm$ 1.0 | 87.1 $\pm$ 0.1 | 87.4 $\pm$ 0.3 | 82.3 $\pm$ 0.5 |
| **AttnSleep** | 78.7 $\pm$ 0.3 | 84.3 $\pm$ 0.3 | 0.785 $\pm$ 0.004 | | 88.5 $\pm$ 0.3 | 46.3 $\pm$ 0.8 | 87.7 $\pm$ 0.2 | 87.8 $\pm$ 0.0 | 83.1 $\pm$ 0.5 |
| **Sleep-FCN** | 77.6 $\pm$ 0.3 | 83.2 $\pm$ 0.3 | 0.772 $\pm$ 0.004 | | 88.7 $\pm$ 0.6 | 43.6 $\pm$ 0.5 | 86.7 $\pm$ 0.2 | 87.7 $\pm$ 0.3 | 81.4 $\pm$ 0.6 |
| **Ours** | **79.8** $\boldsymbol{\pm}$ **0.3** | **84.8** $\boldsymbol{\pm}$ **0.3** | **0.792** $\boldsymbol{\pm}$ **0.004** | | **89.7** $\boldsymbol{\pm}$ **0.8** | **49.4** $\boldsymbol{\pm}$ **1.4** | **87.9** $\boldsymbol{\pm}$ **0.2** | **88.2** $\boldsymbol{\pm}$ **0.2** | **83.6** $\boldsymbol{\pm}$ **0.3** |

**Table S4.** Statistical results of loss weighting methods.

| **Model Comparison** | **Overall metrics** | | | **Class-wise F1-score(%)** | | | | | |
| --- | --- | --- | --- | --- | --- | --- | --- | --- | --- |
|  | **F1-score(%)** | **Accuracy(%)** | **Kappa** | | **Wake** | **N1** | **N2** | **N3** | **REM** |
| **No weighting vs. Ours** | 0.0120 | 0.0162 | 0.0160 | | 0.3098 | 0.0088 | 0.0120 | 0.0006 | 0.0120 |
| **AttnSleep**  **vs. Ours** | 0.1345 | 0.1995 | 0.1989 | | 0.0064 | 0.1345 | 0.3098 | 0.0777 | 0.4227 |
| **Sleep-FCN**  **vs. Ours** | 0.0001 | 0.0001 | 0.0001 | | 0.0543 | 0.0001 | 0.0001 | 0.0088 | 0.0006 |
